# 194. Rapid diagnostic testing to improve management of suspected chlamydia and gonorrhea in adolescents in a Pediatric Emergency Department

**DOI:** 10.1093/ofid/ofac492.272

**Published:** 2022-12-15

**Authors:** Holly M Frost, Michael L Wilson, Genie D Roosevelt

**Affiliations:** Denver Health and Hospital Authority, Denver, Colorado; Denver Health and Hospital Authority, Denver, Colorado; Denver Health and Hospital Authority, Denver, Colorado

## Abstract

**Background:**

Standard turnaround times for *Chlamydia trachomatis* (CT) and *Neisseria gonorrhoeae* (GC) testing result in unnecessary antibiotic use for patients without infection and undertreatment of patients with infection(s). We aimed to determine the impact of rapid CT/GC testing on reducing unnecessary antibiotic use, undertreatment of CT and/or GC, and length of stay in an urban safety-net pediatric emergency department (PED).

**Methods:**

Before 2020, testing for CT/GC was performed using a batched nucleic acid amplification test (NAAT; Hologic Aptima Combo2) with results available the following day. Starting January 2020, the GeneXpert rapid NAAT (Cepheid Xpert CT/NG) with turnaround time between 90-120 minutes was available. Our primary outcome variables were under- and over-treatment. Undertreatment was defined as GC and/or CT positive patients who did not receive appropriate antibiotic treatment in the PED. Overtreatment was defined as GC or CT negative patients who received antibiotic treatment in the PED. Under- and over-treatment percentages were plotted on Statistical Process Control p charts. The balancing measure was length of stay (LOS).

**Results:**

There were 739 patients evaluated in the baseline period (2019), 631 in the intervention period (2020) and 626 in the post-intervention period (2021). There were no differences in gender, race, ethnicity, and insurance across the 3 time periods. After introduction of the GeneXpert, over-treatment decreased from 18.4% to 8.1% (Figure). Under-treatment did not differ. Median turnaround for the GeneXpert was 119 minutes. Median LOS in minutes increased from 165 minutes (baseline) to 182 minutes (intervention) and 202 minutes (post-intervention; p< .001).

Percentage of patients over-treated for chlamydia and gonorrhea.

**Figure:**
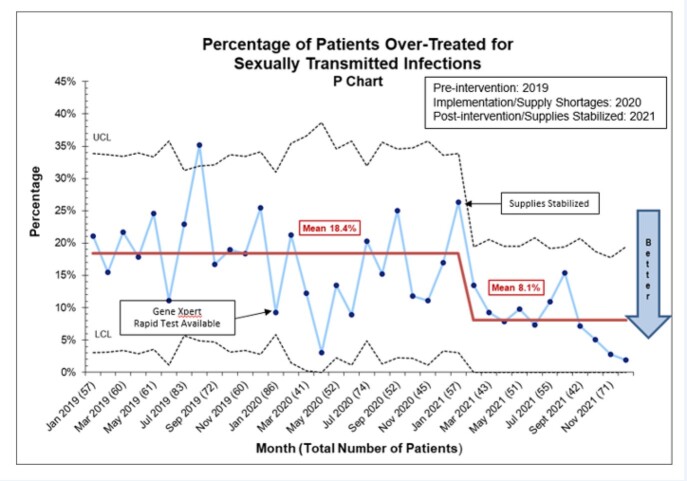

**Conclusion:**

Rapid CT/GC testing substantially reduced unnecessary antibiotic use but increased LOS. Given the rapid increases in CT/GC rates and antimicrobial resistance health systems should consider implementation of rapid testing to appropriately direct antimicrobials to patients most likely to benefit. A more rapid test would likely increase appropriate antibiotic use and limit impact on LOS.

**Disclosures:**

**All Authors**: No reported disclosures.

